# Diferenças entre os Sexos no Infarto Agudo do Miocárdio com Supradesnivelamento do Segmento ST – Análise Retrospectiva de um Único Centro

**DOI:** 10.36660/abc.20211040

**Published:** 2022-12-20

**Authors:** Cátia Costa Oliveira, Filipe Vilela, Carlos Braga, João Costa, Jorge Marques

**Affiliations:** 1 Serviço de Cardiologia Hospital de Braga Braga Portugal Serviço de Cardiologia, Hospital de Braga, Braga – Portugal; 2 Escola de Medicina Universidade do Minho Braga Portugal Escola de Medicina da Universidade do Minho, Braga – Portugal

**Keywords:** Infarto do Miocárdio com Supradesnível do Segmento ST, Mortalidade, Mulheres

## Abstract

**Fundamento:**

Embora os resultados em pacientes com infarto do miocárdio com supradesnivelamento do segmento ST (IAMCSST) submetidos a intervenções coronárias percutâneas (ICP) primárias tenham melhorado, as mulheres apresentam maior mortalidade. Objetivos: Avaliar as diferenças de gênero na apresentação, manejo e mortalidade hospitalar, em 30 dias, 6 meses e 1 ano após IAMCSST.

**Métodos:**

Coletamos retrospectivamente dados de 809 pacientes consecutivos tratados com ICP primária e comparamos mulheres *versus* homens no banco de dados de cardiologia de intervenção local. O nível de significância utilizado foi p<0,05.

**Resultados:**

As mulheres eram mais velhas que os homens (69,1±14,6 vs. 58,5±12,7 anos; p<0,001) com maior prevalência de idade acima de 75 anos (36,7% vs. 11,7%; p<0,001), diabetes (30,6% vs. 18,5%; p=0,001), hipertensão (60,5% vs. 45,9%; p=0,001), doença renal crônica (3,4% vs. 0,6%; p= 0,010) e acidente vascular cerebral isquêmico agudo (6,8% vs. 3,0%; p=0,021). Na apresentação, as mulheres apresentavam mais sintomas atípicos, menos dor torácica (p=0,014) e estavam mais frequentemente em choque cardiogênico (p=0,011)). As mulheres tinham mais tempo até a reperfusão (p=0,001) e eram menos propensas a receber terapia médica ideal (p<0,05). A mortalidade intra-hospitalar (p=0,001), em 30 dias (p<0,001), 6 meses (p<0,001) e 1 ano (16,4% vs. p<0,001) foi maior nas mulheres. A análise multivariada identificou idade acima de 75 anos (HR=4,25; IC 95%[1,67-10,77];p=0,002), classe Killip II (HR=8,80; IC 95%[2,72-28,41];p<0,001), III (HR=5,88; IC95% [0,99-34,80]; p=0,051) e IV (HR=9,60; IC 95%[1,86-48,59];p=0,007), Lesão Renal Aguda (HR=2,47; IC 95% [1,00-6,13];p=0,051) e dias de hospitalização (HR=1,04; IC 95%[1,01-1,08];p=0,030), mas não o sexo feminino (HR=0,83; IC95% [0,33-2,10];p=0,690) como fatores prognósticos independentes de mortalidade.

**Conclusões:**

Comparadas aos homens, as mulheres com IAMCSST submetidas à ICP primária apresentam maiores taxas de mortalidade. Mulheres hospitalizadas por IAMCSST têm pior perfil de risco, são tratadas com maior tempo de reperfusão relacionado a atrasos do sistema e têm menor probabilidade de receber a terapia recomendada. O sexo feminino não foi fator prognóstico independente para mortalidade na população estudada.

## Introdução

Em todo o mundo, a doença arterial coronariana (DAC) está se tornando a principal causa de morte em mulheres.^1 - 5^ Nas últimas décadas, a mortalidade cardiovascular diminuiu devido à melhora na prevenção, diagnóstico e tratamento da doença isquêmica cardíaca. Entretanto, quando comparada aos homens, essa diminuição tem sido menor nas mulheres, principalmente em mulheres mais jovens, com menos de 55 anos.^1 , 3 - 5^

Em relação ao infarto do miocárdio com supradesnivelamento do segmento ST (IAMCSST), existem vários estudos que compararam as diferenças entre os sexos e concluíram que mulheres com IAMCSST apresentam maior risco de morte e eventos adversos após a intervenção coronária percutânea (ICP).^1 , 3 , 5 - 17^ Isso pode ser atribuído à idade mais avançada, à apresentação clínica mais tardia e atípica, à maior prevalência de comorbidades como diabetes mellitus, obesidade, dislipidemia, hipertensão arterial e doença renal crônica (DRC). Entretanto, também tem sido associado ao fato de as mulheres terem menos acesso à tentativa de terapia de revascularização e terapia medicamentosa otimizada.^1 , 3 , 5 - 16^ Ainda é controverso se a maior taxa de mortalidade no sexo feminino é totalmente justificada pela presença de um pior perfil de risco e desigualdades no manejo terapêutico ou se após a análise ajustada de possíveis fatores de confusão, o sexo feminino permanece como fator de risco independente para um pior prognóstico.^7 , 8 , 10 , 14 , 16^

Esse estudo tem como objetivo estudar uma amostra de pacientes com IAMCSST tratados com sucesso com ICP primária, tendo como objetivo comparar os cuidados médicos e os desfechos clínicos, principalmente a mortalidade intra-hospitalar em 30 dias, 6 meses e 1 ano após o IAMCSST, entre homens e mulheres.

## Métodos

### Desenho do estudo

O presente estudo é retrospectivo, enquadrando-se em um estudo observacional analítico e longitudinal. Os dados foram obtidos de um banco de dados de prontuário médico eletrônico. A população estudada incluiu todos os pacientes com diagnóstico de IAMCSST hospitalizados no Hospital de Braga, Portugal, para ICP primária, entre 2013 e 2016. O número total de pacientes, entre janeiro de 2013 e maio de 2016, foi de 834 indivíduos, dos quais 25 foram excluídos após a aplicação dos critérios de inclusão e exclusão definidos abaixo. A análise desse estudo concentrou-se, assim, em 809 pacientes.

#### Critério de inclusão:

- Adultos com diagnóstico de IAMCSST submetidos a ICP primária. Pacientes sem fenômeno de Slow Flow /No Reflow também foram incluídos para fins estatísticos.

#### Critério de exclusão:

- IAMCSST com mais de 12 horas desde o início dos sintomas;
- Pacientes com comorbidades não cardíacas responsáveis por expectativa média de vida esperada <1 ano, documentada antes do início do tratamento;
- Doentes cujos registos informáticos foram impossíveis de consultar;

## Ética

Foram consideradas as regras de Conduta Ética e Boas Práticas, atendendo aos preceitos da Declaração de Helsinque, da Convenção de Direitos Humanos e Biomedicina e às diretrizes do Conselho de Organizações Internacionais de Ciências Médicas. O estudo foi aprovado pela Comissão de Ética para as Ciências da Vida e Saúde da Universidade do Minho e pela Comissão de Ética em Saúde do Hospital de Braga.

## Análise estatística

A análise estatística foi realizada utilizando o software *Statistical Package for the Social Sciences* (SPSS), versão 25, com nível de significância estabelecido em p<0,05. A normalidade das distribuições foi analisada pelo teste de Kolmogorov-Smirnov. A análise descritiva das variáveis qualitativas incluiu a determinação de frequências absolutas e relativas.

O teste do qui-quadrado (χ^2^) e o teste de Fisher foram utilizados para estabelecer associações significantes entre variáveis nominais e ordinais. O primeiro foi utilizado quando não mais que 20% das células da tabela de contingência apresentaram frequência esperada menor que 5; o último foi utilizado nos outros casos.

As variáveis quantitativas foram descritas como média e desvio padrão ou mediana e intervalo interquartil, de acordo com a normalidade dos dados. O teste *t* para amostras independentes e o teste de Mann-Whitney permitiram buscar diferenças em variáveis quantitativas com e sem distribuição normal, respectivamente. O Phi foi calculado como o tamanho do efeito para a associação das variáveis categóricas. O g de Hedge foi o tamanho do efeito calculado para associação de variáveis contínuas. Os limiares considerados foram 0,3 (pequeno), 0,5 (moderado) e 0,8 (grande).

As diferenças de sobrevida entre os sexos durante o primeiro ano após o evento foram analisadas pelo método de Kaplan-Meier e o teste de Log-rank foi usado para avaliar as possíveis diferenças nas curvas de sobrevida dos dois grupos. A regressão multivariada de Cox com seleção *backward* foi usada para determinar preditores independentes de mortalidade durante o primeiro ano após o IAMCSST, após triagem para preditores univariados (p <0,10). Por fim, a validade preditiva do modelo foi avaliada através da curva *Receiver Operating Characteristic* (ROC) e a estabilidade das estimativas foi verificada com o método “bootstrap” para 1.000 amostras.

## Resultados

Um total de 809 pacientes diagnosticados com IAMCSST entre janeiro de 2013 e maio de 2016, foram incluídos no estudo. Os homens constituíram a maioria da amostra (78,1%; n=632). Não foram encontradas diferenças em relação ao IMC ou obesidade. As mulheres eram significativamente mais velhas, com maior prevalência de idade acima de 75 anos e, ao avaliar os fatores de risco cardiovascular, observou-se uma incidência significativamente maior de doença renal crônica (DRC), acidente vascular cerebral isquêmico (AVCi) agudo, doença valvar, diabetes mellitus e hipertensão arterial (nas mulheres, quando comparadas aos homens). Entretanto, a maior frequência de fumantes foi encontrada no sexo masculino ( Tabela 1 ).

**Tabela 1:** Diferenças entres os gêneros

Idade (anos), média±DP	69,1±14,6	58,5±12,7	t=9,44, g=0,80	<,001
**Idade (anos), % (n)**				
>75	36,7 (65)	11,7 (74)	**χ** ^**2**^ **=60,80, φ=0,27**	**<,001**
IMC (kg/m^2^), média±DP	26,5±4,8	27,0±3,8	t=1,26, g=0,12	,210
Obesidade % (n)	18,8 (30)	18,2 (110)	χ^2^=0,07, φ=0,01	,862
**Comorbidades, % (n)**				
DRC	3,4 (6)	0,6 (4)	**χ** ^**2**^ **=8,61, φ=0,10**	**,010** (a)
ICC	1,1 (2)	0,3 (2)	χ^2^=1,86, φ=0,05	,173 (a)
AVCi/AIT	6,8 (12)	3,0 (19)	χ^2^=5,34, φ=0,09	,021
IAM	5,6 (10)	7,3 (46)	χ^2^=0,57, φ=0,03	,451
ICP	3,4 (6)	5,9 (37)	χ^2^=1,67, φ=0,05	,196
CRM	0,0 (0)	0,9 (6)	χ^2^=1,69, φ=0,05	,354 (a)
Doença cardíaca valvular	3,4 (6)	1,1 (7)	**χ** ^**2**^ **=4,55, φ=0,08**	**,044** (a)
DAP	2,8 (5)	4,6 (29)	χ^2^=1,07, φ=0,04	,301
Diabetes mellitus	30,6 (52)	18,5 (110)	**χ** ^**2**^ **=12,52, φ=0,12**	**,001**
Tabagismo	14,0 (24)	66,8 (403)	**χ** ^**2**^ **=150,15, φ=0,44**	**<,001**
Dislipidemia	48,0 (85)	43,0 (271)	χ^2^=1,41, φ=0,04	,236
Hipertensão	60,5 (107)	45,9 (289)	**χ** ^**2**^ **=11,75, φ=0,12**	**,001**
**Sintoma principal, % (n)**				
Dor torácica	90,3 (158)	95,6 (604)	**χ** ^**2**^ **=7,26, φ=0,10**	**<,014** (a)
Parada cardíaca	1,7 (3)	2,7 (17)	χ^2^=0,54, φ=0,03	,591 (a)
Dor epigástrica	3,4 (6)	0,9 (6)	**χ** ^**2**^ **=5,75, φ=0,08**	**,027** (a)
Dispneia	1,7 (3)	0,3 (2)	χ^2^=4,35, φ=0,07	,071 (a)
Síncope	1,1 (2)	0,3 (2)	χ^2^=1,90, φ=0,05	,207 (a)
Náusea	1,1 (2)	0,0 (0)	**χ** ^**2**^ **=7,24, φ=0,10**	**,047** (a)
Escore GRACE, média±DP	134,3±36,3	111,9±30,4	**t=7,42, g=0,70**	**<,001**
**Classe Killip, % (n)**				
I	74,9 (131)	84,7 (535)	**χ** ^**2**^ **=9,12, φ=0,11**	**<,001**
II	14,9 (26)	10,3 (65)	χ^2^=2,80, φ=0,05	,095
III	2,9 (5)	1,1 (7)	χ^2^=2,86, φ=0,06	,091
IV	7,4 (13)	4,0 (25)	χ^2^=3,68, φ=0,07	,055
Choque cardiogênico, % (n)	10,7 (19)	5,4 (34)	**χ** ^**2**^ **=6,47, φ=0,09**	**,011**
Suporte hemodinâmico, % (n)	7,3 (13)	3,6 (23)	**χ** ^**2**^ **=4,47, φ=0,07**	**,035**
Parada cardíaca, % (n)	2,3 (4)	3,0 (19)	χ^2^=0,25, φ=0,02	,799
**Tempo (minutos), Mediana (P25-P75)**				
Início dos sintomas até PCM	95,0 (49,0-180,0)	80,5 (41,0-160,0)	Z=1,24, r=0,04	,215
PCM-até-ECG	13,5 (8,0-37,5)	12,0 (7,0-25,0)	**Z=2,48, r=0,09**	**,013**
Início dos sintomas-até-balão	264,0 (174,0-373,0)	212,5 (153,0-333,0)	**Z=3,23, r=0,11**	**,001**
PCM-até-balão	135,0 (103,0-204,0)	115,0 (86,0-159,5)	**Z=4,48, r=0,16**	**<,001**
**Número de vasos, % (n)**				
1	56,0 (98)	55,2 (344)	χ^2^=0,34, φ=0,01	,854
2	26,3 (46)	29,2 (182)	χ^2^=0,57, φ=0,03	,449
3	17,7 (31)	15,6 (97)	χ^2^=0,47, φ=0,02	,495
Doença do tronco arterial comum, % (n)	7,9 (14)	5,4 (34)	χ^2^=1,48, φ=0,04	,223
**Grau do fluxo TIMI**				
**Pré-ICP, % (n)**				
0	74,4 (131)	66,4 (417)	**χ** ^**2**^ **=4,09, φ=0,07**	**,043**
1	5,7 (10)	6,4 (40)	χ^2^=0,11, φ=0,01	,861
2	10,2 (18)	17,8 (112)	**χ** ^**2**^ **=5,87, φ=0,09**	**,015**
3	9,7 (17)	9,4 (59)	χ^2^=0,01, φ=0,01	,916
**Pós-ICP, % (n)**				
0	0,0 (0)	0,0 (0)	-	-
1	0,0 (0)	0,6 (4)	χ^2^=1,12, φ=0,04	,582 (a)
2	4,6 (8)	3,2 (20)	χ^2^=0,77, φ=0,03	,379
3	95,4 (166)	96,1 (599)	χ^2^=0,20, φ=0,02	,658
**Eventos Adversos, % (n)**				
Parada cardíaca	0,0 (0)	0,5 (3)	χ^2^=0,84, φ=0,03	1,000 (a)
AVCi	0,6 (1)	0,0 (0)	χ^2^=3,58, φ=0,07	,219 (a)
*Slow Flow/No Reflow*	2,8 (5)	0,8 (5)	**χ** ^**2**^ **=4,69, φ=0,08**	**,046**

*TE: tamanho do efeito calculado como g de Hedge para variáveis contínuas e phi (φ) para variáveis categóricas; (a) valor de p calculado com o teste exato de Fisher. DP: desvio padrão; IMC: índice de massa corporal; DRC: doença renal crônica; ICC: insuficiência cardíaca crônica; AVCi/AIT: AVC isquêmico Agudo/Ataque Isquêmico Transitório; IAM: infarto agudo do miocárdio; ICP: intervenção coronária percutânea; CRM: cirurgia de revascularização do miocárdio; DAP: doença arterial periférica. Resultados apresentados como Mediana (P25-P75) devido à alta assimetria. M.W: escore Z do teste de Mann-Whitney; r: tamanho do efeito M.W.; PCM: primeiro contato médico; ECG: eletrocardiograma.*

Em relação à apresentação clínica, a dor torácica esteve mais presente no sexo masculino, aparecendo em 95,6% dos homens contra 90,3% das mulheres. As mulheres apresentaram maior frequência de sintomas atípicos, como dor epigástrica ou náusea, apesar de sua prevalência muito baixa de 3,4% e 1,1% para mulheres e 0,9% e 0,0% para homens, respectivamente. As mulheres também apresentaram mais choque cardiogênico e maior necessidade de suporte hemodinâmico quando comparadas aos homens. O escore GRACE,^1^ uma ferramenta de estratificação de risco cardiovascular e preditor de mortalidade após síndrome coronariana aguda (SCA), também foi significativamente maior no sexo feminino. Por outro lado, a classe 1 de Killip, a classe de melhor prognóstico, mostrou-se mais associada ao sexo masculino (84,7%) do que ao feminino (74,9%) ( Tabela 1 ).

Todos os tempos (em minutos) inerentes ao manejo terapêutico dos pacientes, desde o início dos sintomas até à reperfusão coronária, especificamente o tempo entre o primeiro contato médico (PCM) e a realização do ECG, desde o início dos sintomas até à reperfusão coronária, desde o primeiro contato médico até a reperfusão coronária, foram significativamente maiores em mulheres, exceto pelo tempo entre o início dos sintomas e o primeiro contato médico. ( Tabela 1 )

Também não houve diferenças significantes entre os sexos em relação ao número de vasos acometidos. O fluxo TIMI grau 3 pré-ICP, que representa a obstrução coronária completa, foi mais identificado no sexo feminino (74,4%) quando comparado ao sexo masculino (66,4%) e o fluxo TIMI grau 2 pré-ICP, que representa o atraso do fluxo, mas a perfusão de toda a artéria, foi mais frequentemente identificada no sexo masculino (17,8%) do que no feminino (10,2%), sem associações significantes pós-ICP. A ocorrência de eventos adversos durante a ICP também foi avaliada e verificou-se que o fenômeno *Slow Flow/No Reflow* , embora pouco frequente, associou-se à significância estatística para o sexo feminino ( Tabela 1 ). Em relação à terapia farmacológica instituída na hospitalização, a administração de heparina não fracionada foi significativamente menos prevalente no sexo feminino (85,4%) em relação ao masculino (90,0%). Resultado semelhante foi encontrado durante a ICP com menor uso de heparina não fracionada no sexo feminino. No momento da alta, a prescrição de aspirina, inibidor de P2Y12, betabloqueador e inibidores da enzima conversora de angiotensina (IECA) foi significativamente menor nas mulheres. Por outro lado, antagonistas de vitamina K (AVK), controle glicêmico com insulina e antidiabéticos orais foram maiores no sexo feminino ( Tabela 2 ).

**Tabela 2:** Terapia farmacológica na hospitalização, durante a ICP e na alta hospitalar

Variável %, (n)	Na hospitalização				Durante a ICP				Na alta hospitalar			
		
	Feminino (n=177)	Masculino (n=632)	χ^**2**^, T.E.	p-valor	Feminino (n=177)	Masculino (n=632)	χ^**2**^, T.E.	p-valor	Feminino (n=160)	Masculino (n=610)	χ^**2**^, T.E.	p-valor
Aspirina	96,6 (171)	98,7 (624)	χ^2^=3,67, φ=0,07	,094 (a)	4,5 (8)	2,2 (14)	χ^2^=2,78, φ=0,06	,115 (a)	93,1 (149)	99,2 (605)	**χ** ^**2**^ **=22,84, φ=0,17**	**<,001** (a)
iP2Y12	93,3 (165)	95,3 (602)	χ^2^=1,16, φ=0,04	,281	6,67 (12)	4,43 (28)	χ^2^=1,38, φ=0,04	,240	91,9 (147)	98,2 (599)	**χ** ^**2**^ **=16,78, φ=0,15**	**<,001** (a)
HBPM	14,6 (22)	9,9 (57)	χ^2^=1,83, φ=0,05	,212	12,3 (19)	8,0 (46)	χ^2^=2,01, φ=0,05	,157	N/A	N/A	N/A	N/A
HNF	85,4 (129)	90,0 (520)	**χ** ^**2**^ **=7,70, φ=0,10**	**,006**	87,7 (135)	92,0 (530)	**χ** ^**2**^ **=5,70, φ=0,08**	**,017**	N/A	N/A	N/A	N/A
Eptifibatide	N/A	N/A	N/A	N/A	11,1 (3)	9,2 (12)	χ^2^=0,03, φ=0,01	>,990	N/A	N/A	N/A	N/A
Abciximabe	N/A	N/A	N/A	N/A	22,2 (6)	28,2 (37)	χ^2^=1,67, φ=0,05	,196	N/A	N/A	N/A	N/A
Tirofibana	N/A	N/A	N/A	N/A	66,7 (18)	62,6 (82)	χ^2^=1,00, φ=0,04	,316 (a)	N/A	N/A	N/A	N/A
NAOs	N/A	N/A	N/A	N/A	N/A	N/A	N/A	N/A	13,0 (3)	18,2 (8)	χ^2^=0,29, φ=0,02	,706
AVK	N/A	N/A	N/A	N/A	N/A	N/A	N/A	N/A	87,0 (20)	81,8 (36)	**χ** ^**2**^ **=8,18, φ=0,10**	**,004** (a)
Betabloqueadores	N/A	N/A	N/A	N/A	N/A	N/A	N/A	N/A	90,6 (145)	95,1 (580)	**χ** ^**2**^ **=4,58, φ=0,08**	**,032**
IECA	N/A	N/A	N/A	N/A	N/A	N/A	N/A	N/A	88,1 (141)	94,8 (578)	**χ** ^**2**^ **=9,01, φ=0,11**	**,003**
BRAs	N/A	N/A	N/A	N/A	N/A	N/A	N/A	N/A	5,6 (9)	2,6 (16)	χ^2^=3,64, φ=0,07	,057
Estatinas	N/A	N/A	N/A	N/A	N/A	N/A	N/A	N/A	97,5 (156)	99,2 (605)	χ^2^=3,10, φ=0,06	,095 (a)
ADO	N/A	N/A	N/A	N/A	N/A	N/A	N/A	N/A	18,6 (30)	12,0 (73)	**χ** ^**2**^ **=5,03, φ=0,08**	**,025**
Insulina	N/A	N/A	N/A	N/A	N/A	N/A	N/A	N/A	9,3 (15)	1,0 (6)	**χ** ^**2**^ **=33,64, φ=0,21**	**<,001** (a)

*T.E.: tamanho do efeito calculado como phi (φ) para variáveis categóricas; (a) valor de p calculado com teste exato de Fisher. iP2Y12: inibidor de P2Y12; HBPM: heparina de baixo peso molecular; HNF: heparina não fracionada; NAOs: novos anticoagulantes orais; AVK: antagonista da vitamina k; IECA: inibidores da enzima conversora de angiotensina; BRAs: bloqueadores dos receptores da angiotensina II; ADO: antidiabéticos orais.*

Durante a hospitalização ( Tabela 3 ), verificou-se que as mulheres apresentaram maior chance de evolução clínica desfavorável, representada por uma classe Killip mais grave quando comparada aos homens, com quase o dobro das mulheres na classe Killip II, com diferença ainda maior em relação para a classe Killip III e classe Killip IV. Por outro lado, a classe Killip I foi novamente mais frequentemente associada ao sexo masculino.

**Tabela 3:** Características clínicas e complicações intra-hospitalares

Variável	Gênero		Estatística, T.E.	p-valor

	Feminino (n=177)	Masculino (n=632)		
**FEVE, % (n)**				
Preservada	39,8 (66)	39,2 (242)	χ^2^=0,02, φ=0,01	,900
Leve	25,3 (42)	26,6 (164)	χ^2^=0,11, φ=0,01	,740
Moderada	25,3 (42)	26,6 (164)	χ^2^=0,11, φ=0,01	,740
Grave	9,6 (16)	7,6 (47)	χ^2^=0,72, φ=0,03	,395
Troponina I, Mediana (P25-P75)	69,8 (26,8-132,0)	74,9 (33,5-138,0)	Z=0,96, r=0,04	,340
**Classe Killip, % (n)**				
I	57,7 (101)	78,0 (490)	**χ** ^**2**^ **=29,06, φ=0,19**	**<,001**
II	26,3 (46)	15,1 (95)	**χ** ^**2**^ **=11,77, φ=0,12**	**<,001**
III	6,3 (11)	1,8 (11)	**χ** ^**2**^ **=10,56, φ=0,12**	**<,001** (a)
IV	9,7 (17)	5,1 (32)	**χ** ^**2**^ **=5,10, φ=0,08**	**,024**
**Eventos Cardiovasculares, % (n)**				
Re-IM	1,1 (2)	1,6 (10)	χ^2^=0,19, φ=0,02	>,990 (a)
Sangramento maior	0,6 (1)	1,1 (7)	χ^2^=0,42, φ=0,02	>,990 (a)
AVCi/AIT	2,8 (5)	1,6 (10)	χ^2^=1,20, φ=0,04	,339 (a)
LRA	11,4 (20)	4,0 (25)	**χ** ^**2**^ **=14,37, φ=0,13**	**<,001**
Parada cardíaca	9,0 (16)	5,9 (37)	χ^2^=2,29, φ=0,05	,130
Complicações mecânicas	0,6 (1)	1,1 (7)	χ^2^=0,28, φ=0,02	>,990 (a)
IC	30,7 (54)	18,4 (116)	**χ** ^**2**^ **=12,59, φ=0,13**	**,001**
FA	10,7 (19)	5,7 (36)	**χ** ^**2**^ **=5,54, φ=0,08**	**,019**
CRM	0,0 (0)	1,3 (8)	χ^2^=2,26, φ=0,05	,212 (a)
Dias de hospitalização, Mediana (P25-P75)	5,0 (4,5-7,0)	4,0 (4,0-5,0)	**Z=4,34, r=0,15**	**<,001**

*T.E.: tamanho do efeito calculado como phi (φ) para variáveis categóricas; M.W.: escore Z do teste de Mann-Whitney; r = tamanho do efeito de M.W.; Resultados apresentados como Mediana (P25-P75) devido à alta assimetria; (a) Teste exato de Fisher. FEVE: fração de ejeção do ventrículo esquerdo; IM: infarto do miocárdio; AVCi/AIT: AVC isquêmico agudo/ataque isquêmico transitório; LRA: lesão renal aguda; IC: insuficiência cardíaca; FA: fibrilação atrial; CRM: cirurgia de revascularização do miocárdio.*

A pior evolução durante a hospitalização em mulheres também resultou no desenvolvimento de complicações pós-infarto mais frequentes, como Lesão Renal Aguda, Insuficiência Cardíaca e Fibrilação Atrial, com escore mediano de um dia a mais de hospitalização do que os homens.

A Tabela 4 mostra a análise comparativa da mortalidade por gênero, em diferentes períodos de tempo, ao longo do primeiro ano após a ocorrência do IAMCSST. O sexo feminino mostrou-se associado a maior mortalidade em todos os momentos, maior mortalidade intra-hospitalar, em 30 dias, após 6 meses e mortalidade de 1 ano.

**Tabela 4:** Mortalidade e eventos cardiovasculares durante 1 ano após IAMCSST

Variável	Gênero		χ^**2**^, T.E.	p-valor

	Feminino (n=177)	Masculino (n=632)		
**Mortalidade, % (n)**				
Intra-hospitalar	9,6 (17)	3,5 (22)	**χ** ^**2**^ **=11,30, φ=0,12**	**,001**
30 dias	11,3 (20)	4,0 (25)	**χ** ^**2**^ **=14,20, φ=0,13**	**<,001**
6 meses	14,1 (25)	4,7 (30)	**χ** ^**2**^ **=19,19, φ=0,15**	**<,001**
1 ano	16,4 (29)	6,3 (40)	**χ** ^**2**^ **=17,92, φ=0,15**	**<,001**
**Eventos cardiovasculares**				
Rehospitalização	11,3 (18)	7,9 (48)	χ^2^=1,85, φ=0,05	,174
IAM	2,5 (4)	1,3 (8)	χ^2^=1,17, φ=0,04	>,990 (a)
AVCi	2,5 (4)	0,3 (2)	**χ** ^**2**^ **=7,74, φ=0,10**	**,019 (a)**
ICP	5,6 (9)	6,7 (41)	χ^2^=0,26, φ=0,02	,613
CRM	1,3 (2)	0,8 (5)	χ^2^=0,26, φ=0,02	,640 (a)

*T.E.: tamanho do efeito calculado como phi (φ) para variáveis categóricas; (a) valor de p calculado com teste exato de Fisher. CV: cardiovascular; IAMCSST: infarto agudo do miocárdio com supradesnivelamento do segmento ST; IAM: infarto agudo do miocárdio; AVCi: AVC isquêmico agudo; ICP: intervenção coronária percutânea; CRM: cirurgia de revascularização do miocárdio.*

Em relação à ocorrência de novos eventos cardiovasculares, durante o primeiro ano após o evento, foram observadas diferenças significantes para AVCi com maior prevalência do sexo feminino (2,5%) em relação ao masculino (0,3%).

**Figura 1:**
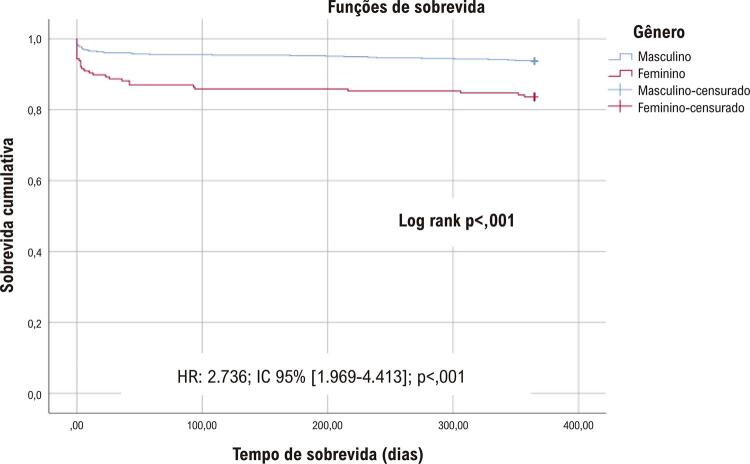
Análise de sobrevida de Kaplan-Meier.

Na análise de sobrevida pelo método de Kaplan-Meier ( Figura 1 ), as curvas de sobrevida masculina e feminina foram representadas durante o primeiro ano após o IAMCSST. A distribuição de sobrevida avaliada pelo teste de Log-rank (Mantel-Cox) foi significativamente diferente entre os dois grupos.

Após o IAMCSST, as mulheres tiveram uma taxa de mortalidade 2,7 vezes maior do que os homens.

O conjunto final de análise objetivou avaliar a resposta multivariada do gênero quando ajustada a diversas outras variáveis relevantes. Essas variáveis foram então analisadas para significância marginal (p<0,10) relacionada à mortalidade no primeiro ano após a ocorrência de IAMCSST.

Em seguida, todas as variáveis marginais significantes foram incluídas em um modelo multivariado, seguindo o método Enter. Quando ajustado para todas essas variáveis, o sexo feminino não apresentou significância estatística. Assim, realizamos uma análise exploratória tentando encontrar o modelo mais parcimonioso, ou seja, aquele com o número mais adequado de variáveis, visando manter uma alta capacidade preditiva. Seguindo um algoritmo de etapas, incluímos na primeira etapa da regressão multivariada de Cox todas as variáveis com p<0,10 e, em seguida, iniciou-se um processo de seleção *backward* , removendo consecutivamente todas as variáveis sem relevância estatística. Os resultados da etapa final ( Tabela 5 ) mostraram maior chance de mortalidade para pacientes com mais de 75 anos; a classe Killip na hospitalização revelou um risco aumentado estatisticamente significante de mortalidade para as classes II e IV e um risco aumentado marginalmente significante de mortalidade para a classe III. A LRA intra-hospitalar revelou associação marginal com a mortalidade. Por fim, a chance de mortalidade tende a aumentar em 4% a cada dia de hospitalização ( Tabela 5 ).

**Tabela 5:** Análise de regressão de Cox univariada e multivariada

Variável	Univariada		Multivariada (seleção *backward* ) 15º passo		

	HR	p-valor	HR	IC95%	p-valor
Gênero feminino	**2,74**	**p<,001**	-	-	-
Idade (> 75)	**6,15**	**p<,001**	**4,25**	**1,67 – 10,77**	**p=,002**
DRC	**5,49**	**p<,001**	-	-	-
AVCi/AIT	1,57	p=,385	-	-	-
DM	**2,13**	**p=,004**	-	-	-
Classe Killip na hospitalização					
I	1	1	1	1	1
II	**7,14**	**p<,001**	**8,80**	**2,72 – 28,41**	**p<,001**
III	2,24	p=,430	**5,88**	**0,99 – 34,80**	**p=,051**
IV	**15,23**	**p<,001**	**9,60**	**1,86 – 48,59**	**p=,007**
Choque cardiogênico na hospitalização	**10,37**	**p<,001**	-	-	-
Tempo de início dos sintomas-até-balão	**1,01**	**p<,001**	-	-	-
Tempo do PMC-até- balão	**1,01**	**p<,001**	-	-	-
Grau de fluxo TIMI pré-ICP					
0	**8,41**	**p=,035**	-	-	-
1	3,09	p=,357	-	-	-
2	4,83	p=,138	-	-	-
3	1	1	-	-	-
Terapia na alta hospitalar					
Aspirina	**0,09**	**p<,001**	-	-	-
iP2Y12	**0,15**	**p<,001**	-	-	-
Betabloqueadores	0,40	p=,089	-	-	-
IECA	1,00	p>,990	-	-	-
BRAs	0,05	p=,502	-	-	-
FEVE					
Preservada	1	1	-	-	-
Leve	1,84	p=,175	-	-	-
Moderada	**2,39**	**p=,042**	-	-	-
Grave	**9,27**	**p<,001**	-	-	-
Classe Killip intra-hospitalar					
I	1	1	-	-	-
II	**14,53**	**p<,001**	-	-	-
III	**38,44**	**p<,001**	-	-	-
IV	**73,82**	**p<,001**	-	-	-
Complicações intra-hospitalares					
LRA	**5,74**	**p<,001**	**2,47**	**1,00 - 6,13**	**p=,051**
Parada cardíaca	**11,55**	**p<,001**	-	-	-
IC	**4,57**	**p<,001**	-	-	-
FA	**2,42**	**p=,010**	-	-	-
Dias de hospitalização	**1,07**	**p<,001**	**1,04**	**1,01 – 1,08**	**p=,030**

*HR: Hazard Ratio; IC95% CI: intervalo de confiança de 95%; DRC: doença renal crônica; AVCi/AIT: AVC isquêmico agudo/ataque isquêmico transitório; DM: diabetes mellitus; PCM: primeiro contato médico; iP2Y12: inibidor de P2Y12; IECA: inibidores da enzima conversora de angiotensina; BRAs: bloqueadores dos receptores da angiotensina II; FEVE: fração de ejeção do ventrículo esquerdo; LRA: lesão renal aguda; IC: insuficiência cardíaca; FA: fibrilação atrial.*

**Figura 2:**
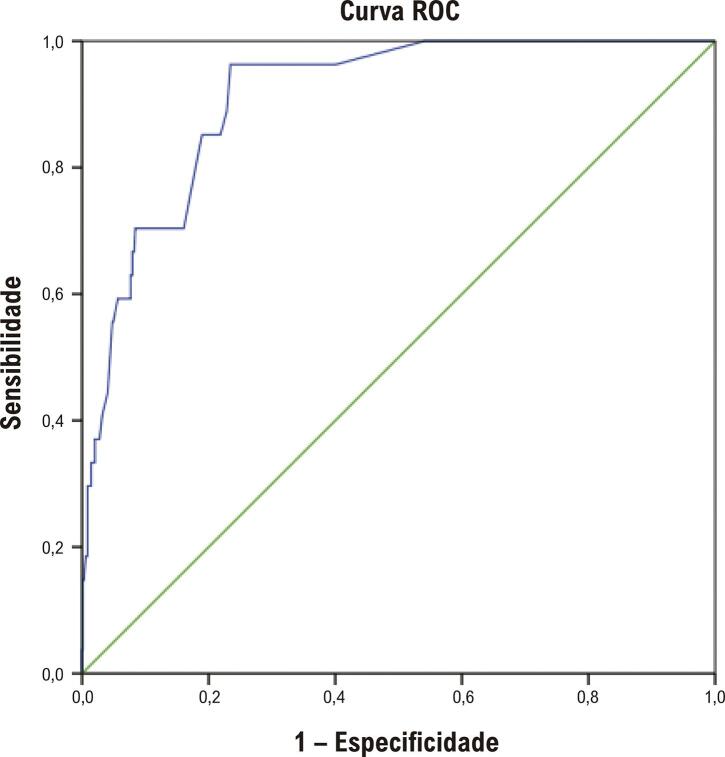
Avaliação da curva ROC para a capacidade do modelo preditivo.

A curva ROC apresentou uma capacidade preditiva muito alta, com área sob a curva (AUC, *Area Under the Curve* ) de 91,0% ( Figura 2 ). Essa área (“modelo *backward* ”) foi apenas ligeiramente inferior à do “modelo Enter”, confirmando que a opção mais parcimoniosa foi a mais adequada.

Finalmente, uma análise utilizando o método *bootstrap* de 1.000 amostras foi realizada para avaliar a validade do modelo. Uma amostra *bootstrap* é obtida por amostragem com reposição, nesse caso 1.000 vezes, visando avaliar a precisão das estimativas e sua respectiva significância. Os resultados do *bootstrap* não mostraram evidência de viés significativo e confirmaram a significância estatística dos fatores de risco idade >75 anos, Killip classe II ou III na hospitalização, LRA intra-hospitalar e dias de internação. Entretanto, isso descartou a análise do impacto da classe Killip na hospitalização^4^ ( Tabela 6 ).

**Tabela 6:** Análise de *bootstrap* para o modelo final

Variáveis	Viés	*EP*	p-valor
Idade (> 75a)	0,02	0,56	**p<,001**
Classe Killip na hospitalização			
I	1	1	1
II	0,01	0,51	**p=,049**
III	0,16	0,82	**p=,001**
IV	0,36	2,07	p=,266
LRA intra-hospitalar	0,05	0,51	**p=,014**
Dias de hospitalização	-0,01	0,02	**p<,001**

*LRA: lesão renal aguda.*

## Discussão

A mortalidade cardiovascular diminuiu nas últimas décadas, devido ao progresso na prevenção, diagnóstico e tratamento da doença isquêmica cardíaca; entretanto, as doenças cardiovasculares continuam sendo a principal causa de morte em países desenvolvidos, com maior mortalidade no sexo feminino, principalmente em pacientes com IAMCSST.

A maioria dos estudos realizados nesse contexto são claros, isto é, há uma maior taxa de mortalidade nas mulheres quando comparadas aos homens. No entanto, os autores não são unânimes em explicar esse achado, com três fatores principais que podem justificar a maior mortalidade em mulheres.

Em primeiro lugar, as características biológicas e as diferenças fisiopatológicas inerentes ao sexo feminino têm sido sugeridas para justificar a maior mortalidade em mulheres quando comparadas aos homens. Um dos fatores potenciais é o estrogênio, conhecido como protetor do sistema cardiovascular, cujos valores diminuem significativamente em mulheres na pós-menopausa. Além disso, a fisiopatologia da DAC tem características distintas nas mulheres, especificamente doença aterosclerótica menos difusa, sendo a dissecção espontânea das artérias coronárias, síndrome de Takotsubo ou disfunção endotelial mais frequente nas mulheres.^8 , 12 , 15^ Entretanto, quando possíveis fatores de confusão são levados em consideração, as conclusões permanecem controversas; há autores que identificam o sexo feminino como fator prognóstico independente para mortalidade, enquanto outros não o fazem.^7 , 8 , 10 , 14 , 16^

Em segundo lugar, o pior prognóstico em mulheres tem sido atribuído ao pior perfil cardiovascular. Elas geralmente são mais velhas, com maior frequência de diabetes mellitus, hipertensão arterial, DRC ou acidente vascular cerebral.^5 - 8 , 12 , 15^

Por fim, a assistência médica desigual, como o maior tempo de isquemia devido ao atraso entre o início dos sintomas e a reperfusão, bem como a menor probabilidade de terapia médica otimizada de prevenção secundária, também têm sido apontada como uma das causas do aumento da mortalidade em mulheres com IAMCSST.^5 - 8 , 12 , 15^

Este estudo demonstrou que, como descrito na literatura, mulheres com diagnóstico de IAMCSST submetidas a ICP primária apresentam pior perfil cardiovascular quando comparadas aos homens. Elas são frequentemente mais velhas na apresentação, com maior prevalência de idade acima de 75 anos, têm mais diabetes mellitus e hipertensão arterial. Além disso, as mulheres têm uma história cardiovascular mais desfavorável, com as mulheres apresentando maior prevalência de DRC, acidente vascular cerebral e doença valvar.^5 , 7 , 8 , 10 , 11^ Em relação à apresentação clínica, as mulheres apresentaram maior frequência de sintomas atípicos, especificamente dor epigástrica ou náusea, ao invés da clássica dor torácica, mais frequente no sexo masculino. Essas informações foram descritas de forma robusta na literatura e podem afetar negativamente o diagnóstico e a abordagem terapêutica, o que pode contribuir para atrasos na revascularização e apresentação clínica mais grave.^6 , 7 , 17 , 18^

Em relação ao tempo necessário para a reperfusão coronariana, as mulheres foram consecutivamente prejudicadas em relação aos homens, apresentando maior tempo entre o início dos sintomas e ICP, entre PCM e ECG ou PCM e ICP e, posteriormente, maior grau de isquemia miocárdica.^5 , 7 , 8 , 12 , 14 , 17^

O único momento em que não houve diferenças significantes entre os dois gêneros foi entre o início dos sintomas e o PCM, o que aponta para o fato de que a maior demora no atendimento das mulheres não é atribuída à desvalorização dos sintomas pelos pacientes, mas, possivelmente, ao sistema de saúde.^18^ Isso pode ser explicado, em parte, pela apresentação clínica atípica e maior dificuldade diagnóstica ou por alguma desvalorização da prevalência dessa patologia no sexo feminino.^5 - 8 , 14 , 17 , 18^

O maior perfil de risco cardiovascular e a desigualdade quanto ao tempo de reperfusão podem explicar o fato de as mulheres apresentarem mais choque cardiogênico na apresentação, representado por maior escore GRACE, mais choque cardiogênico e necessidade mais frequente de suporte hemodinâmico.^8 , 10^

Em relação às características angiográficas, nosso estudo não demonstrou diferenças significantes entre os dois gêneros, seja na frequência de doença multiarterial ou doença do tronco arterial comum.^7 , 10 , 17^ No entanto, durante a ICP, é importante destacar a apresentação com pior grau de fluxo TIMI (grau 0) e maior ocorrência do fenômeno *Slow Flow/No Reflow* em mulheres, outros fatores que podem contribuir para maior grau de isquemia miocárdica e pior prognóstico.

Também mostramos que as mulheres têm maior probabilidade de evolução clínica desfavorável, ou seja, mais dias de hospitalização, classe Killip mais grave e desenvolvimento de complicações pós-IAMCSST mais frequentes, como Lesão Renal Aguda, Insuficiência Cardíaca e Fibrilação Atrial.^5 , 8 , 11^

Em relação à terapia farmacológica implementada, de acordo com os resultados publicados, nosso estudo corroborou que as mulheres são menos propensas a serem medicadas com terapia medicamentosa otimizada quando comparadas aos homens. As mulheres receberam heparina não fracionada com menos frequência na hospitalização e menos aspirina, inibidores de P2Y12, betabloqueador ou IECA na alta hospitalar, o que pode contribuir para um pior prognóstico em longo prazo.^5 , 11^

Após a análise multivariada, o sexo feminino não mostrou ser um fator prognóstico independente para mortalidade após IAMCSST; por outro lado, idade acima de 75 anos, maior classe Killip na hospitalização, LRA intra-hospitalar e dias de internação foram identificados como fatores associados ao aumento do risco de mortalidade, com o modelo apresentando uma capacidade preditiva muito alta (AUC = 91,0% - p<0,001), sendo importante ressaltar que todos esses fatores foram identificados com maior frequência nas mulheres.

### Limitações

Este estudo apresenta algumas limitações importantes: foi um estudo observacional, não cego e não-randomizado, podendo, portanto, estar sujeito a fatores de confusão que não foram reconhecidos ou levados em consideração na análise dos dados. Em segundo lugar, os dados são derivados de um único centro, o que limita sua aplicabilidade.

## Conclusões

Como nosso estudo demonstra que o sexo feminino não é um fator prognóstico independente para mortalidade após IAMCSST, podemos concluir que a maior mortalidade entre as mulheres não se deve à fragilidade biológica relacionada ao sexo. Portanto, a maior mortalidade das mulheres pode ser explicada pelo fato de as mulheres serem mais velhas, apresentarem pior perfil cardiovascular, apresentarem maior disfunção ventricular esquerda na hospitalização, refletida pela classe Killip mais grave, maior grau de isquemia miocárdica causada pelo maior tempo de reperfusão coronária, secundário a atrasos do sistema, bem como uma menor frequência de terapia médica otimizada.
