# Supplementary material for: Diferenças entre os Sexos no Infarto Agudo do Miocárdio com Supradesnivelamento do Segmento ST – Análise Retrospectiva de um Único Centro
Source: Arq Bras Cardiol. 2022 Dec 20;120(1):e20211040. [Article in Portuguese] doi: 10.36660/abc.20211040 (PMC9833215; doi:10.36660/abc.20211040)
Supplement: Translation

# Translation: ST-Segment Elevation Myocardial Infarction Differences between Genders – A Single Center Retrospective Analysis

**Authors:** Cátia Costa Oliveira, Filipe Vilela, Carlos Braga, João Costa, Jorge Marques

**Affiliations:** 1 Portugal Serviço de Cardiologia, Hospital de Braga, Braga – Portugal; 2 Portugal Escola de Medicina da Universidade do Minho, Braga – Portugal

**Keywords:** ST Elevation Myocardial Infarction, Mortality, Women

## Abstract

**Background:**

Although outcomes in patients with ST-segment elevation myocardial infarction (STEMI) undergoing primary percutaneous coronary interventions (PCI) have improved, women show higher mortality.

**Objectives:**

To assess gender differences in presentation, management and in-hospital mortality, at 30-days, 6-months and 1-year after STEMI.

**Methods:**

We retrospectively collected data from 809 consecutive patients treated with primary PCI and compared the females versus males at the local intervention cardiology database. The level of significance used was p<0.05.

**Results:**

Women were older than man (69,1±14,6 vs. 58,5±12,7 years; p<.001) with higher prevalence of age over 75 years (36.7% vs. 11.7%; p<.001), diabetes (30,6% vs. 18,5%; p=.001), hypertension (60.5% vs. 45.9%; p=.001), chronic kidney disease (3.4% vs. 0.6%; p=.010) and acute ischemic stroke (6.8% vs. 3.0%; p=.021). At presentation, women had more atypical symptoms, less chest pain (p=.014) and were more frequently in cardiogenic shock (p=.011)). Women had longer time until reperfusion (p=.001) and were less likely to receive optimal medical therapy (p<0.05). In-hospital mortality (p=.001), at 30-days (p<.001), 6-months (p<.001) and 1-year (16.4% vs. p<.001) was higher in women. The multivariate analysis identified age over 75 years (HR=4.25; 95% CI[1.67-10.77];p=.002), Killip class II (HR=8.80; 95% CI[2.72-28.41];p<.001), III (HR=5.88; 95% CI [0.99-34.80]; p=.051) and IV (HR=9.60; 95% CI[1.86-48.59];p=.007), Acute Kidney Injury (HR=2.47; 95% CI[1.00-6.13];p=.051) and days of hospitalization (HR=1.04; 95% CI[1.01-1.08];p=.030) but not female gender (HR=0.83; 95% CI[0.33-2.10];p=.690) as independent prognostic factors of mortality.

**Conclusions:**

Compared to men, women with STEMI undergoing primary PCI have higher mortality rates. Women admitted for STEMI have a worse risk profile, are treated with a higher reperfusion time related with system delays and are less likely to receive the recommended therapy. Female gender was not an independent prognostic factor for mortality in the studied population.

## Introduction

Worldwide, coronary artery disease (CAD) is becoming the leading cause of death in women.^1 - 5^In the last decades, cardiovascular mortality has decreased due to the improvement of prevention, diagnosis and treatment of ischemic heart disease.

However, when compared to men, this decrease has been smaller in women, especially in younger women, under 55 years old.^1 , 3 - 5^

Regarding ST-segment elevation myocardial infarction (STEMI), there have been several studies that compared the differences between genders and concluded that women with STEMI have a higher risk of death and adverse events after PCI.^1 , 3 , 5 - 17^This can be attributed to the older age, the later and atypical clinical presentation, the higher prevalence of comorbidities such as diabetes mellitus, obesity, dyslipidemia, arterial hypertension and chronic kidney disease (CKD). However, it has also been associated with the fact that women have less access to attempted revascularization therapy and optimized medical therapy.^1 , 3 , 5 - 16^It is still controversial whether the higher mortality rate in the female gender is totally justified by the presence of worse risk profile and inequalities in therapeutic management or if after adjusted analysis of possible confounding factors, the female gender remains an independent risk factor for worse prognostic.^7 , 8 , 10 , 14 , 16^

This work aims to study a sample of patients with STEMI successfully treated with primary PCI, having as its goal to compare the medical care and clinical outcomes, especially in-hospital mortality at 30-days, 6-months and 1-year after the STEMI, between men and women.

## Methods

### Study design

The present study is retrospective, fitting in an analytical and longitudinal observational study. The data were derived from an electronic health record database. The studied population included all patients diagnosed with STEMI admitted at Hospital de Braga, Portugal, for primary PCI, between 2013 and 2016. The total number of patients, between January 2013 and May 2016, comprised 834 individuals, of which 25 were excluded after the implementation of the inclusion and exclusion criteria defined below. The analysis of this study thus focused on 809 patients.

#### Inclusion Criteria:

- Adults diagnosed with STEMI who underwent primary PCI. Patients with slow flow/ no reflow phenomenon were also included for statistical purposes.

#### Exclusion Criteria:

- STEMI with more than 12 hours since symptom onset;
- Patients with non-cardiac comorbidities responsible for an expected average life expectancy <1 year, documented prior to the start of the treatment;
- Patients whose computer records were impossible to check;

## Ethics

The rules of Ethical Conduct and Good Practice were taken into consideration, complying with the precepts of the Helsinki Declaration, the Human Rights and Biomedicine Convention and the guidelines of the Council for International Medical Sciences Organizations. The study was approved by the Committee on Ethics for Life Sciences and Health of Universidade do Minho and the Health Ethics Committee of Hospital de Braga.

## Statistical analysis

The statistical analysis was performed using the Statistical Package for the Social Sciences (SPSS), version 25, with a significance level set at p<0.05. The normality of the distributions was analyzed using the Kolmogorov-Smirnov test. The descriptive analysis of qualitative variables included the determination of absolute and relative frequencies.

The chi-square (χ^2^) test and Fisher’s test were used to establish significant associations between nominal and ordinal variables. The former was used when no more than 20% of the contingency table cells had an expected frequency lower than 5; the last was used otherwise.

Quantitative variables were described as mean and standard deviation or median and interquartile range, according to data normality. The t-test for independent samples and the Mann-Whitney test allowed us to look for differences in quantitative variables with and without normal distribution, respectively. Phi was calculated as the effect size for categorical variables’ association. Hedges’ g was the effect size calculated for continuous variables’ association. The thresholds considered were 0.3 (small), 0.5 (moderate) and 0.8 (large).

Differences in survival between genders during the first year after the event were analyzed using the Kaplan-Meier method and the Log-rank test was used to evaluate the possible differences in the survival curves of the two groups. Multivariate Cox regression with a Backward selection was used to determine independent predictors of mortality during the first year after STEMI, after screening for univariate predictors (p<.10). Finally, the predictive validity of the model was assessed using the Receiver Operating Characteristic (ROC) curve and the stability of the estimates was verified by conducting a 1,000 sample bootstrap.

## Results

A total of 809 patients diagnosed with STEMI between January 2013 and May 2016, were included in the study. Men constituted the majority of the sample (78,1%; n=632). No differences were found regarding BMI or obesity. Women were significantly older, with a higher prevalence of age over 75 years and, when assessing cardiovascular risk factors, there was a significantly higher incidence of chronic kidney disease (CKD), acute ischemic stroke (AIS), Valvular disease, Diabetes mellitus and Hypertension (in women, when compared to men). However, the highest frequency of smokers was found in males ( Table 1 ).

**Table 1:** Gender differences

Age (years), mean±SD	69.1±14.6	58.5±12.7	t=9.44, g=0.80	<.001
**Age (years), % (n)**				
>75	36.7 (65)	11.7 (74)	**χ** ^**2**^ **=60.80, φ=0.27**	**<.001**
BMI (kg/m^2^), mean±SD	26.5±4.8	27.0±3.8	t=1.26, g=0.12	.210
Obesity % (n)	18.8 (30)	18.2 (110)	χ^2^=0.07, φ=0.01	.862
**Comorbidities, % (n)**				
CKD	3.4 (6)	0.6 (4)	**χ** ^**2**^ **=8.61, φ=0.10**	**.010** (a)
CHF	1.1 (2)	0.3 (2)	χ^2^=1.86, φ=0.05	.173 (a)
AIS/TIA	6.8 (12)	3.0 (19)	χ^2^=5.34, φ=0.09	.021
AMI	5.6 (10)	7.3 (46)	χ^2^=0.57, φ=0.03	.451
PCI	3.4 (6)	5.9 (37)	χ^2^=1.67, φ=0.05	.196
CABG	0.0 (0)	0.9 (6)	χ^2^=1.69, φ=0.05	.354 (a)
Valvular heart disease	3.4 (6)	1.1 (7)	**χ** ^**2**^ **=4.55, φ=0.08**	**.044** (a)
PAD	2.8 (5)	4.6 (29)	χ^2^=1.07, φ=0.04	.301
Diabetes mellitus	30.6 (52)	18.5 (110)	**χ** ^**2**^ **=12.52, φ=0.12**	**.001**
Smoking	14.0 (24)	66.8 (403)	**χ** ^**2**^ **=150.15, φ=0.44**	**<.001**
Dyslipidemia	48.0 (85)	43.0 (271)	χ^2^=1.41, φ=0.04	.236
Hypertension	60.5 (107)	45.9 (289)	**χ** ^**2**^ **=11.75, φ=0.12**	33,2 mm
**Main Symptom, % (n)**				
Chest pain	90.3 (158)	95.6 (604)	**χ** ^**2**^ **=7.26, φ=0.10**	**<.014** (a)
Cardiac arrest	1.7 (3)	2.7 (17)	χ^2^=0.54, φ=0.03	.591 (a)
Epigastric pain	3.4 (6)	0.9 (6)	**χ** ^**2**^ **=5.75, φ=0.08**	.027 (a)
Dyspnea	1.7 (3)	0.3 (2)	χ^2^=4.35, φ=0.07	.071 (a)
Syncope	1.1 (2)	0.3 (2)	χ^2^=1.90, φ=0.05	.207 (a)
Nausea	1.1 (2)	0.0 (0)	**χ** ^**2**^ **=7.24, φ=0.10**	**.047 (a)**
GRACE score, mean±SD	134.3±36.3	111.9±30.4	**t=7.42, g=0.70**	**<.001**
**Killip class, % (n)**				
I	74.9 (131)	84.7 (535)	**χ** ^**2**^ **=9.12, φ=0.11**	**<.001**
II	14.9 (26)	10.3 (65)	χ^2^=2.80, φ=0.05	.095
III	2.9 (5)	1.1 (7)	χ^2^=2.86, φ=0.06	.091
IV	7.4 (13)	4.0 (25)	χ^2^=3.68, φ=0.07	.055
Cardiogenic shock, % (n)	10.7 (19)	5.4 (34)	**χ** ^**2**^ **=6.47, φ=0.09**	**.011**
Hemodynamic support, % (n)	7.3 (13)	3.6 (23)	**χ** ^**2**^ **=4.47, φ=0.07**	**.035**
Cardiac arrest, % (n)	2.3 (4)	3.0 (19)	χ^2^=0.25, φ=0.02	.799
**Time (minutes), Mdn (P25-P75)**				
Symptom onset-to-FMC	95.0 (49.0-180.0)	80.5 (41.0-160.0)	Z=1.24, r=0.04	.215
FMC-to-ECG	13.5 (8.0-37.5)	12.0 (7.0-25.0)	**Z=2.48, r=0.09**	**.013**
Symptom onset-to-balloon	264.0 (174.0-373.0)	212.5 (153.0-333.0)	**Z=3.23, r=0.11**	**.001**
FMC-to-balloon	135.0 (103.0-204.0)	115.0 (86.0-159.5)	**Z=4.48, r=0.16**	**<.001**
**Number of vessels, % (n)**				
1	56.0 (98)	55.2 (344)	χ^2^=0.34, φ=0.01	.854
2	26.3 (46)	29.2 (182)	χ^2^=0.57, φ=0.03	.449
3	17.7 (31)	15.6 (97)	χ^2^=0.47, φ=0.02	.495
Left main coronary disease, % (n)	7.9 (14)	5.4 (34)	χ^2^=1.48, φ=0.04	.223
**TIMI flow grade**				
**Pre-PCI, % (n)**				
0	74.4 (131)	66.4 (417)	**χ** ^**2**^ **=4.09, φ=0.07**	**.043**
1	5.7 (10)	6.4 (40)	χ^2^=0.11, φ=0.01	.861
2	10.2 (18)	17.8 (112)	**χ** ^**2**^ **=5.87, φ=0.09**	**.015**
3	9.7 (17)	9.4 (59)	χ^2^=0.01, φ=0.01	.916
**Post-PCI, % (n)**				
0	0.0 (0)	0.0 (0)	-	-
1	0,0 (0)	0.6 (4)	χ^2^=1.12, φ=0.04	.582 (a)
2	4.6 (8)	3.2 (20)	χ^2^=0.77, φ=0.03	.379
3	95.4 (166)	96.1 (599)	χ^2^=0.20, φ=0.02	.658
**Adverse Events, % (n)**				
Cardiac arrest	0.0 (0)	0.5 (3)	χ^2^=0.84, φ=0.03	1.000 (a)
AIS	0.6 (1)	0.0 (0)	χ^2^=3.58, φ=0.07	.219 (a)
Slow Flow/No Reflow	2.8 (5)	0.8 (5)	**χ** ^**2**^ **=4.69, φ=0.08**	**.046**

*ES: Effect size calculated as Hegde’s g for continuous variables and phi (φ) for categorical ones; (a) p-value calculated with Fisher’s exact test. SD: standard deviation; BMI: body mass index; CKD: chronic kidney disease; CHF: chronic heart failure; AIS/TIA: acute ischemic stroke/transient ischemic attack; AMI: acute myocardial infarction; PCI: percutaneous coronary intervention; CABG: coronary artery bypass graft; PAD: *peripheral artery disease* . Results presented as Median (Mdn) (P25-P75) due to high skewness. M.W.: Mann-Whitney test Z score; r: M.W. effect size; FMC: first medical contact; ECG: electrocardiogram; PCI: percutaneous coronary intervention.*

In relation to clinical presentation, chest pain was more frequently present in males, appearing in 95.6% of the men against 90.3% of the women. Women showed a higher frequency of atypical symptoms, such as epigastric pain or nausea, despite their very low prevalence of 3.4% and 1.1% for women and 0.9% and 0.0% for men, respectively. Women also had more cardiogenic shock and greater need for hemodynamic support when compared to men. The GRACE score, a cardiovascular risk stratification tool and predictor of mortality after acute coronary syndrome (ACS), was also significantly higher in females. On the other hand, Killip class 1, the best prognosis class, was found to be more associated with males (84.7%) than females (74.9%) ( Table 1 ).

All the times (in minutes) inherent to the therapeutic management of patients, from symptom onset to coronary reperfusion, namely the time between the first medical contact (FMC) and the performance of the ECG, from symptom onset to coronary reperfusion, from the first medical contact to coronary reperfusion, were significantly higher in women, except for the time between symptom onset and the first medical contact ( Table 1 ).

There were no significant differences between genders in relation to the number of affected vessels, either. TIMI grade 3 flow pre-PCI, which represents complete coronary obstruction, was more often identified in females (74.4%) when compared to males (66.4%) and TIMI flow grade 2 pre-PCI, which represents delayed flow but perfusion of entire artery, was more often identified in males (17.8%) than females (10.2%), with no significant associations post-PCI. The occurrence of adverse events during PCI was also evaluated and it was verified that the Slow Flow/No Reflow phenomenon, although infrequent, was associated with statistical significance to the female gender ( Table 1 ).

In relation to the established pharmacological therapy at admission, the administration of unfractionated heparin was significantly less prevalent in females (85.4%) compared to males (90.0%). A similar result was found during PCI with less use of unfractionated heparin in females. At the time of discharge, the prescription of aspirin, P2Y12 inhibitor, beta-blocker and ACEIs was significantly lower in women. On the other hand, VKA, glycemic control with insulin and oral antidiabetics were higher in females ( Table 2 ).

**Table 2:** Pharmacological therapy at admission, during PCI and at hospital discharge

Variable %, (n)	At admission				During PCI				At discharge			
		
	Female (n=177)	Male (n=632)	χ^**2**^, E.S.	p-value	Female (n=177)	Male (n=632)	χ^**2**^, E.S.	p-value	Female (n=160)	Male (n=610)	χ^**2**^, E.S.	p-value
Aspirin	96.6 (171)	98.7 (624)	χ^2^=3.67, φ=0.07	.094 (a)	4.5 (8)	2.2 (14)	χ^2^=2.78, φ=0.06	.115 (a)	93.1 (149)	99.2 (605)	**χ** ^**2**^ **=22.84, φ=0.17**	**<.001** (a)
iP2Y12	93.3 (165)	95.3 (602)	χ^2^=1.16, φ=0.04	.281	6.67 (12)	4.43 (28)	χ^2^=1.38, φ=0.04	.240	91.9 (147)	98.2 (599)	**χ** ^**2**^ **=16.78, φ=0.15**	**<.001** (a)
LMWH	14.6 (22)	9.9 (57)	χ^2^=1.83, φ=0.05	.212	12.3 (19)	8.0 (46)	χ^2^=2.01, φ=0.05	.157	N/A	N/A	N/A	N/A
UFH	85.4 (129)	90.0 (520)	**χ** ^**2**^ **=7.70, φ=0.10**	**.006**	87.7 (135)	92.0 (530)	**χ** ^**2**^ **=5.70, φ=0.08**	**.017**	N/A	N/A	N/A	N/A
Eptifibatide	N/A	N/A	N/A	N/A	11.1 (3)	9.2 (12)	χ^2^=0.03, φ=0.01	>.990	N/A	N/A	N/A	N/A
Abciximab	N/A	N/A	N/A	N/A	22.2 (6)	28.2 (37)	χ^2^=1.67, φ=0.05	.196	N/A	N/A	N/A	N/A
Tirofiban	N/A	N/A	N/A	N/A	66.7 (18)	62.6 (82)	χ^2^=1.00, φ=0.04	.316 (a)	N/A	N/A	N/A	N/A
NOAC	N/A	N/A	N/A	N/A	N/A	N/A	N/A	N/A	13.0 (3)	18.2 (8)	**χ** ^**2**^ **=0.29, φ=0.02**	**.706**
VKA	N/A	N/A	N/A	N/A	N/A	N/A	N/A	N/A	87.0 (20)	81.8 (36)	**χ** ^**2**^ **=8.18, φ=0.10**	**.004** (a)
Beta-blockers	N/A	N/A	N/A	N/A	N/A	N/A	N/A	N/A	90.6 (145)	95.1 (580)	**χ** ^**2**^ **=4.58, φ=0.08**	**.032**
ACEIs	N/A	N/A	N/A	N/A	N/A	N/A	N/A	N/A	88.1 (141)	94.8 (578)	χ^2^=9.01, φ=0.11	.003
ARBs	N/A	N/A	N/A	N/A	N/A	N/A	N/A	N/A	5.6 (9)	2.6 (16)	χ^2^=3.64, φ=0.07	.057
Statin	N/A	N/A	N/A	N/A	N/A	N/A	N/A	N/A	97.5 (156)	99.2 (605)	χ^2^=3.10, φ=0.06	.095 (a)
OAD	N/A	N/A	N/A	N/A	N/A	N/A	N/A	N/A	18.6 (30)	12.0 (73)	**χ** ^**2**^ **=5.03, φ=0.08**	**.025**
Insulin	N/A	N/A	N/A	N/A	N/A	N/A	N/A	N/A	9.3 (15)	1.0 (6)	**χ** ^**2**^ **=33.64, φ=0.21**	**<.001** (a)

*ES: Effect size calculated as phi (φ) for categorical variables; (a) p-value calculated with Fisher exact test. iP2Y12: P2Y12 inhibitor; LMWH: low molecular weight heparin; UFH: unfractionated heparin; NOAC: novel oral anticoagulants; VKA: vitamin k antagonist; ACEIs: angiotensin-converting enzyme inhibitors; ARBs: angiotensin II receptor blockers; OAD: oral antidiabetic drugs.*

During hospitalization ( Table 3 ), it was found that women were more likely to have unfavorable clinical progression, represented by a more severe Killip class when compared to men, with nearly twice the women in Killip class II, with an even greater difference in relation to Killip class III and Killip class IV. On the other hand, Killip class I was again more frequently associated with males.

**Table 3:** Clinical characteristics and in-hospital complications

Variable	Gender		Statistic, E.S.	p-value

	Female (n=177)	Male (n=632)		
**LVEF, % (n)**				
Preserved	39.8 (66)	39.2 (242)	χ^2^=0.02, φ=0.01	.900
Mild	25.3 (42)	26.6 (164)	χ^2^=0.11, φ=0.01	.740
Moderate	25.3 (42)	26.6 (164)	χ^2^=0.11, φ=0.01	.740
Severe	9.6 (16)	7.6 (47)	χ^2^=0.72, φ=0.03	.395
Troponin I, Mdn (P25-P75)	69.8 (26.8-132.0)	74.9 (33.5-138.0)	Z=0.96, r=0.04	.340
**Killip class, % (n)**				
I	57.7 (101)	78.0 (490)	**χ** ^**2**^ **=29.06, φ=0.19**	**<.001**
II	26.3 (46)	15.1 (95)	**χ** ^**2**^ **=11.77, φ=0.12**	**<.001**
III	6.3 (11)	1.8 (11)	**χ** ^**2**^ **=10.56, φ=0.12**	**<.001** (a)
IV	9.7 (17)	5.1 (32)	**χ** ^**2**^ **=5.10, φ=0.08**	**.024**
**Cardiovascular Events, % (n)**				
Re-MI	1.1 (2)	1.6 (10)	χ^2^=0.19, φ=0.02	>.990 (a)
Major bleeding	0.6 (1)	1.1 (7)	χ^2^=0.42, φ=0.02	>.990 (a)
AIS/TIA	2.8 (5)	1.6 (10)	χ^2^=1.20, φ=0.04	.339 (a)
AKI	11.4 (20)	4.0 (25)	**χ** ^**2**^ **=14.37, φ=0.13**	**<.001**
Cardiac arrest	9.0 (16)	5.9 (37)	χ^2^=2.29, φ=0.05	.130
Mechanical complications	0.6 (1)	1.1 (7)	χ^2^=0.28, φ=0.02	>.990 (a)
HF	30.7 (54)	18.4 (116)	**χ** ^**2**^ **=12.59, φ=0.13**	**.001**
AF	10.7 (19)	5.7 (36)	**χ** ^**2**^ **=5.54, φ=0.08**	**.019**
CABG	0.0 (0)	1.3 (8)	χ^2^=2.26, φ=0.05	.212 (a)
Hospitalization days, Mdn (P25-P75)	5.0 (4.5-7.0)	4.0 (4.0-5.0)	**Z=4.34, r=0.15**	**<.001**

*ES: Effect size calculated as phi (φ) for categorical variables; M.W.: Mann-Whitney test Z score; r: M.W. effect size; Results presented as Median (Mdn) (P25-P75) due to high skewness; (a) Fisher exact test. LVEF: left ventricular ejection fraction; MI: myocardial infarction; AIS/TIA: acute ischemic stroke/transitory ischemic attack; AKI: acute kidney injury; HF: heart failure; AF: atrial fibrillation; CABG: coronary artery bypass graft.*

The worse progression during hospitalization in women also resulted in the development of more frequent post-infarct complications, such as Acute Kidney Injury, Heart Failure and Atrial Fibrillation, with a median score of one more day of hospitalization than men.

Table 4 shows the comparative analysis of gender mortality, in different periods of time, over the first year after the occurrence of STEMI. Female gender proved to be associated with higher mortality at all times, higher in-hospital mortality, within 30-days, after 6-month mortality and 1-year mortality.

**Table 4:** Mortality and Cardiovascular Events during 1 year after STEMI

Variable	Gender		χ^**2**^, E.S.	p-value

	Female (n=177)	Male (n=632)		
Mortality, % (n)				
In-hospital	9.6 (17)	3.5 (22)	χ^2^=11.30, φ=0.12	.001
30 day	11.3 (20)	4.0 (25)	χ^2^=14.20, φ=0.13	<.001
6 month	14.1 (25)	4.7 (30)	χ^2^=19.19, φ=0.15	<.001
1 year	16.4 (29)	6.3 (40)	χ^2^=17.92, φ=0.15	<.001
Cardiovascular Events				
Hospital Readmission	11.3 (18)	7.9 (48)	χ^2^=1.85, φ=0.05	.174
AMI	2.5 (4)	1.3 (8)	χ^2^=1.17, φ=0.04	>.990 (a)
AIS	2.5 (4)	0.3 (2)	χ^2^=7.74, φ=0.10	.019 (a)
PCI	5.6 (9)	6.7 (41)	χ^2^=0.26, φ=0.02	.613
CABG	1.3 (2)	0.8 (5)	χ^2^=0.26, φ=0.02	.640 (a)

*ES: effect size calculated as phi (φ) for categorical variables; (a) p-value calculated with Fisher exact test. CV: cardiovascular; STEMI: ST-segment elevation myocardial infarction; AMI: acute myocardial infarction; AIS: acute ischemic stroke; PCI: percutaneous coronary intervention; CABG: coronary artery bypass graft.*

Regarding the occurrence of new cardiovascular events, during the first year after the event, significant differences were observed for AIS with higher prevalence of females (2.5%) compared to males (0.3%).

In the survival analysis using the Kaplan-Meier method ( Figure 1 ), the male and female survival curves were represented during the first year after STEMI. The survival distribution evaluated by the Log-rank test (Mantel-Cox) was significantly different between the two groups.

**Figure 1:** Kaplan-Meier survival analysis.

After the STEMI, women had a 2.7-fold higher mortality rate than men.

The final set of analysis intended to assess the multivariate response of gender when adjusted to several other relevant variables. These variables were then screened for marginal significance (p<0.10) related to mortality over the first year after the occurrence of STEMI.

Then, all marginal significant variables were included in a multivariate model following the Enter method. When adjusted for all these variables, female gender did not show any statistical significance. Hence, we performed an exploratory analysis attempting to find the most parsimonious model, i.e. with the optimal number of variables, aiming to maintain a high predictive capacity. By following a step algorithm, we included in the first step of the multivariate Cox regression all variables with p<0.10 and then a backward selection process began, consecutively removing all variables without statistical relevance. The final step results ( Table 5 ) showed an increased chance of mortality for patients older than 75 years; the Killip class at admission revealed a statistically significant increased risk of mortality for classes II and IV and marginally significant increased risk of mortality for class III. In-hospital AKI revealed a marginal association with mortality. Finally, the chance of mortality tends to increase by 4% for each hospitalization day ( Table 5 ).

**Table 5:** Univariate and Multivariate Cox regression analysis

Variable	Univariate		Multivariate (Backward selection) 15^**th**^step		
	
	HR	p-value	HR	95% CI	p-value
Female gender	**2.74**	**p<.001**	-	-	-
Age (> 75)	**6.15**	**p<.001**	**4.25**	**1.67 – 10.77**	**p=.002**
CKD	**5.49**	**p<.001**	-	-	-
AIS/TIA	1.57	p=.385	-	-	-
DM	**2.13**	**p=.004**	-	-	-
Killip class at admission					
I	1	1	1	1	1
II	**7.14**	**p<.001**	**8.80**	**2.72 – 28.41**	**p<.001**
III	2.24	p=.430	**5.88**	**0.99 – 34.80**	**p=.051**
IV	15.23	p<.001	**9.60**	**1.86 – 48.59**	**p=.007**
Cardiogenic shock at admission	**10.37**	**p<.001**	-	-	-
Symptom onset-to-balloon time	**1.01**	**p<.001**	-	-	-
FMC-to-balloon time	**1.01**	**p<.001**	-	-	-
Pre-PCI TIMI flow grade					
0	**8.41**	**p=.035**	-	-	-
1	3.09	p=.357	-	-	-
2	4.83	p=.138	-	-	-
3	1	1	-	-	-
Therapy at discharge					
Aspirin	0.09	p<.001	-	-	-
iP2Y12	0.15	p<.001	-	-	-
Beta-blockers	0.40	p=.089	-	-	-
ACEIs	1.00	p>.990	-	-	-
ARBs	0.05	p=.502	-	-	-
LVEF					
Preserved	1	1	-	-	-
Mild	1.84	p=.175	-	-	-
Moderate	**2.39**	**p=.042**	-	-	-
Severe	**9.27**	**p<.001**	-	-	-
In-hospital Killip class					
I	1	1	-	-	-
II	**14.53**	**p<.001**	-	-	-
III	**38.44**	**p<.001**	-	-	-
IV	**73.82**	**p<.001**	-	-	-
In-hospital complications					
AKI	**5.74**	**p<.001**	**2.47**	**1.00 - 6.13**	**p=.051**
Cardiac arrest	**11.55**	**p<.001**	-	-	-
HF	**4.57**	**p<.001**	-	-	-
AF	**2.42**	**p=.010**	-	-	-
Hospitalization days	**1.07**	**p<.001**	**1,04**	**1.01 – 1.08**	**p=.030**

*HR: hazard ratio; 95% CI: 95% confidence interval; CKD: chronic kidney disease; AIS/TIA: acute ischemic stroke/transitory ischemic attack; DM: diabetes mellitus; FMC: first medical contact; iP2Y12: P2Y12 inhibitor; ACEIs: angiotensin-converting enzyme inhibitors; ARBs: angiotensin II receptor blockers; LVEF: left ventricular ejection fraction; AKI: acute kidney injury; HF: heart failure; AF: atrial fibrillation.*

The ROC curve showed a very high predictive capacity, with an area under the curve (AUC) of 91.0% ( Figure 2 ). This area (“backward model”) was just slightly lower than the one of the “Enter model”, confirming that the most parsimonious option was more adequate.

**Figure 2:** ROC curve assessment for the predictive model capacity.

Finally, a 1000 samples bootstrap analysis was performed to assess model validity. A bootstrap sample is obtained by sampling with replacement, in this case 1000 times, aiming to assess the precision of the estimates and their respective significance. Bootstrap results showed no evidence for significant bias and confirmed the statistical significance of the risk factors age > 75, Killip class 2 or 3 at admission, in-hospital AKI and hospitalization days. Nevertheless, this dismissed the analysis of the impact of Killip class at admission^4^( Table 6 ).

**Table 6:** Bootstrap analysis for final model

Variables	Bias	*SE*	p-value
Age (> 75)	0.02	0.56	**p<.001**
Killip class at admission			
I	1	1	1
II	0.01	0.51	**p=.049**
III	0.16	0.82	**p=.001**
IV	0.36	2.07	**p=.266**
In-hospital AKI	0.05	0.51	**p=.014**
Hospitalization days	-0.01	0.02	**p<.001**

*AKI: acute kidney injury.*

## Discussion

Cardiovascular mortality has decreased in recent decades, due to the progress in prevention, diagnosis and treatment of ischemic heart disease; however, cardiovascular diseases remain the leading cause of death in developed countries, with higher mortality in the female gender, mainly in patients with STEMI.

Most of the studies carried out in this context are clear, i.e., there is a higher mortality rate in women when compared to men. However, the authors are not unanimous in explaining this finding, with three main factors that may justify the higher mortality in women.

First, the biological characteristics and pathophysiological differences inherent to the female gender have been suggested to justify higher mortality in women when compared to men. One of the potential factors is estrogen, known as a protector of the cardiovascular system, whose values decrease significantly in postmenopausal women. In addition, the pathophysiology of CAD in women has distinct characteristics, namely less diffuse atherosclerotic disease, with spontaneous dissection of the coronary arteries, Takotsubo syndrome or endothelial dysfunction being more frequent in women.^8 , 12 , 15^Nevertheless, when possible confounding factors are taken into account, the conclusions remain controversial; there are authors who identify the female gender as an independent prognostic factor for mortality, while others do not.^7 , 8 , 10 , 14 , 16^

Second, the worst prognosis in women has been attributed to the worse cardiovascular profile. Usually, they are older, with higher frequency of diabetes mellitus, arterial hypertension, CKD or stroke.^5 - 8 , 12 , 15^

Finally, the unequal medical care, such as the longer ischemia time due to the delay between symptom onset and reperfusion, as well as the lower probability of optimized secondary prevention medical therapy have also been identified as one of the causes for the increased mortality in women with STEMI.^5 - 8 , 12 , 15^

This study demonstrated that, as described in the literature, women diagnosed with STEMI undergoing primary PCI have a worse cardiovascular profile when compared to men. They are often older at presentation, with higher prevalence of age over 75 years, they have more diabetes mellitus and arterial hypertension. Moreover, females have a more unfavorable cardiovascular history, with women having a higher prevalence of CKD, stroke and valvular disease.^5 , 7 , 8 , 10 , 11^Regarding the clinical presentation, women had a higher frequency of atypical symptoms, namely epigastric pain or nausea, rather than the classic chest pain, more frequent in males. This information has been robustly described in literature and may negatively affect the diagnosis and therapeutic approach, which may contribute to delays in revascularization and more severe clinical presentation.^6 , 7 , 17 , 18^

Regarding the time required for coronary reperfusion, women were consecutively impaired in relation to men, showing longer time between the symptom onset and PCI, between FMC and ECG or FMC and PCI and, subsequently a higher degree of myocardial ischemia.^5 , 7 , 8 , 12 , 14 , 17^

The only time in which there were no significant differences between the two genders, was between the onset of symptoms and FMC, which points to the fact that the greater delay in treating women is not attributed to the devaluation of symptoms by patients but, possibly, to the health system.^18^This may partly be explained by the atypical clinical presentation and greater diagnostic difficulty or by some devaluation of the prevalence of this pathology in the female gender.^5 - 8 , 14 , 17 , 18^

The higher cardiovascular risk profile and the inequality regarding the time to reperfusion may explain the fact that women were more frequently in cardiogenic shock at presentation, represented by a higher GRACE score, more cardiogenic shock and the more frequent need for hemodynamic support.^8 , 10^

In relation to the angiographic characteristics, our study demonstrated no significant differences between the two genders, whether in the frequency of multivessel disease or common arterial trunk disease.^7 , 10 , 17^However, during the PCI, it is important to highlight the presentation with worse TIMI flow grade (grade 0) and the higher occurrence of the Slow Flow/No Reflow phenomenon in women, other factors that may contribute to a higher degree of myocardial ischemia and worse prognosis.

We also showed that women have a higher probability of unfavorable clinical progression, namely, more hospitalization days, more severe Killip class and the development of more frequent post-STEMI complications, such as Acute Kidney Injury, Heart Failure and Atrial Fibrillation.^5 , 8 , 11^

Regarding the implemented pharmacological therapy, in accordance with published results, our study corroborated that women are less likely to be medicated with optimized medical therapy when compared to men. Women received unfractionated heparin less frequently at admission and less aspirin, P2Y12 inhibitors, beta-blocker or ACEIs at discharge, which may contribute to a worse long-term prognosis.^5 , 11^

After the multivariate analysis, the female gender has not shown to be an independent prognostic factor for mortality after STEMI; on the other hand, age over 75 years, higher Killip class at admission, in-hospital AKI and days of hospitalization were identified as factors associated with increased risk of mortality, with the model showing a very high predictive capacity (AUC = 91.0% (p<.001)), and it is important to emphasize that all of these factors were more frequently identified in women.

### Limitations

This study has some important limitations: it was an observational, unblinded and non-randomized study, and may therefore be subject to confounding factors that were not recognized or taken into account in the data analysis. Secondly, the data are derived from a single center, which limits their applicability.

## Conclusions

Since our study demonstrate that the female gender is not an independent prognostic factor for mortality after STEMI, we can conclude that the higher mortality among women is not due to gender-related biological fragility. Therefore, the higher mortality of women can be explained by the fact that women were older, had a worse cardiovascular profile, had greater left ventricular dysfunction at admission reflected by more severe Killip class, a higher degree of myocardial ischemia caused by the longer coronary reperfusion time secondary to system delays, as well as a lower frequency of optimized medical therapy.
